# Binding and Conversion of Selenium in *Candida utilis* ATCC 9950 Yeasts in Bioreactor Culture

**DOI:** 10.3390/molecules22030352

**Published:** 2017-02-25

**Authors:** Marek Kieliszek, Stanisław Błażejak, Eliza Kurek

**Affiliations:** 1 Faculty of Food Sciences, Department of Biotechnology, Microbiology and Food Evaluation, Warsaw University of Life Sciences—SGGW, Nowoursynowska 159 C, 02-776 Warsaw, Poland; stanislaw_blazejak@sggw.pl; 2 Faculty of Chemistry, Biological and Chemical Research Centre, University of Warsaw, Zwirki i Wigury 101, 02-089 Warsaw, Poland; kurek@chem.uw.edu.pl

**Keywords:** selenium, selenomethionine, *Candida utilis*, yeast, bioreactor

## Abstract

Selenium is considered an essential component of all living organisms. The use of yeasts as a selenium supplement in human nutrition has gained much interest over the last decade. The accumulation and biochemical transformation of selenium in yeast cells is particularly interesting to many researchers. In this article, we present the results of the determination of selenium and selenomethionine content in the biomass of feed yeast *Candida utilis* ATCC 9950 obtained from the culture grown in a bioreactor. The results indicated that *C. utilis* cells performed the biotransformation of inorganic selenium(IV) to organic derivatives (e.g., selenomethionine). Selenium introduced (20–30 mg Se^4+^∙L^−1^) to the experimental media in the form of sodium(IV) selenite (Na_2_SeO_3_) salt caused a significant increase in selenium content in the biomass of *C. utilis*, irrespective of the concentration. The highest amount of selenium (1841 μg∙g_d.w._^−1^) was obtained after a 48-h culture in media containing 30 mg Se^4+^∙L^−1^. The highest content of selenomethionine (238.8 μg∙g_d.w._^−1^) was found after 48-h culture from the experimental medium that was supplemented with selenium at a concentration of 20 mg Se^4+^∙L^−1^. Biomass cell in the cultures supplemented with selenium ranged from 1.5 to 14.1 g∙L^−1^. The results of this study indicate that yeast cell biomass of *C. utilis* enriched mainly with the organic forms of selenium can be a valuable source of protein. It creates the possibility of obtaining selenium biocomplexes that can be used in the production of protein-selenium dietary supplements for animals and humans

## 1. Introduction

Selenium is vital to the proper functioning of an organism; it exhibits antioxidant properties and protects an organism against free radicals and carcinogens. The protective role of selenium against pro-oxidants is, inter alia, due to the presence of selenium in the active center of various antioxidant enzymes [1]. It also ensures the proper functioning of the thyroid gland [2]. It enhances immunity and demonstrates detoxification properties, and also protects an organism against heavy metals. Thus, selenium deficiency can cause many diseases, including neurological diseases, Keshan disease, or Kashin–Beck disease [3].

In nature, selenium is present in two forms: organic and inorganic. The inorganic form of selenium can be found in various minerals, as exemplified by selenite, selenate, and elementary selenium. Organic forms of selenium can be detected in food products as an integral part of various organic compounds, including amino acids such as selenomethionine (SeMet) and selenocysteine (SeCys). SeCys is obtained primarily from products of animal origin, whereas SeMet can be obtained from plant products such as cereals, legumes, and green leafy vegetables [4]. Thus, the primary source of selenium is the diet. Although selenium present in food and feed is varied, the most valuable and concurrently safe methods of supplementation include selenium yeast preparations enriched with organic forms of selenium. Their application exhibits multidirectional beneficial effects on human health [5].

Due to its chemical similarity to sulfur, selenium metabolism takes place via the same or similar pathway. Selenium amino acids—primarily SeMet—can get incorporated into proteinsnon-specifically by replacing sulfur amino acids. Thus they constitute a stored reserve of selenium that is used by an organism in contrast to other forms of selenium that are subjected to continuous exchange (selenite-exchangeable metabolic pool, Se-EMP). The most absorbable form of selenium is l-SeMet. d-SeMet is about five-fold less well-absorbed than l-SeMet, and is metabolized primarily via the decomposition of selenium present in the inorganic form [6]. SeMet is decomposed to alanine and hydrogen selenide (H_2_Se) with the participation of the enzyme selenocysteine-β-lyase [7]. Both isomers of SeMet must be converted to SeCys during the conversion to H_2_Se [8].

Compared to inorganic forms, the bioavailability of selenium in the form of SeMet is highest in the case of humans and animal organisms [3]. This is due to the non-specific binding of methionine observed in proteins of higher organisms [9]. SeMet is efficiently stored in various tissues, where it exhibits antioxidant properties, improves immunity, stimulates the activity of DNA repair enzymes, and so on [10,11].

Yeasts can uptake selenium ions from the environment and then stably incorporate them in their cell structure. Selenium yeasts are the primary dietary supplement containing organic forms of selenium [12]. Depending on the culture conditions, yeasts can accumulate large amounts of selenium (3000 μg∙g_d.w._^−1^) [13,14]. The high degree of selenium uptake and eventual transformation into organic forms justifies the possible use of yeast cell biomass for the production of selenium supplements. According to a study by Gharieb and Gadd [15], SeMet is the main form of selenium in yeast cells, but it depends on culture conditions and the yeast strain.

In this study, we aimed to evaluate the possibility of the intracellular binding of selenium by *Candida utilis* ATCC 9950 feed yeasts in media supplemented with sodium(IV) selenite (Na_2_SeO_3_) salt in the bioreactor culture, and to determine the content of selenium and SeMet in the resulting yeast cell biomass.

## 2. Results and Discussion

### 2.1. Yeast Cell Biomass Yield

**Figure 1:**
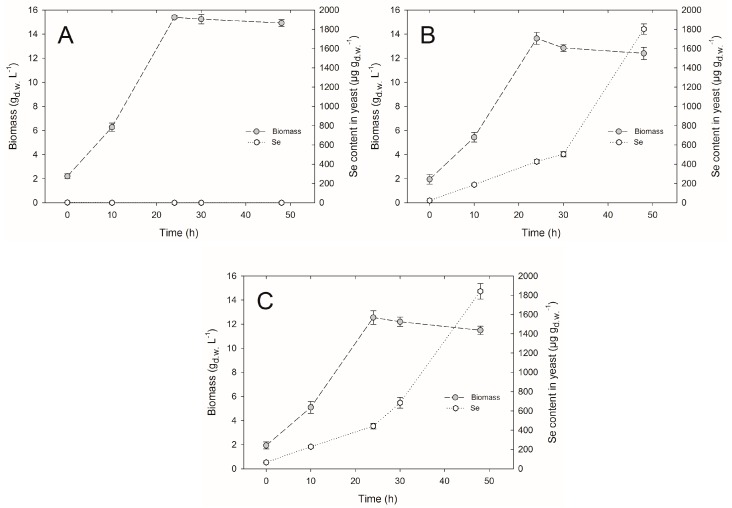
The culture of *Candida utilis* ATCC 9950 yeasts in (**A**) the control (yeast extract peptone dextrose, YPD) and in the experimental media enriched with selenium at (**B**) 20 mg∙L^−1^ and (**C**) 30 mg∙L^−1^.

The volumetric productivity of biomass is one of the main factors determining the profitability of biotechnological processes involving microorganisms. Sodium salts of selenium negatively affected the growth of *C. utilis* ATCC 9950 yeasts. Under the influence of selenite and with an extension of culture time for *C. utilis*, yeast biomass concentration was lower compared to the yield obtained from the control culture. After the introduction of inoculum to the control yeast extract peptone dextrose (YPD) medium (without selenium addition), the yield of cell biomass was 2.1 g_d.w._∙L^−1^, whereas it reached the value of 15.3 g_d.w._∙L^−1^ in 24-h culture (Figure 1A). Prolonged time of culture up to 30 and 48 h resulted in a decrease in the cell biomass by 0.1 and 0.4 g_d.w._ L^−1^, respectively. Statistical analysis demonstrated that during the second day of growth, the yeast biomass concentration was not subjected to a significant decrease compared to the 24-h culture. In the case of experimental cultures, the highest biomass yield (amounting to 13.6 g_d.w._∙L^−1^) was obtained at a concentration of 20 mg Se^4+^∙L^−1^, and after 24 h (Figure 1B). Statistical analysis also revealed that the resulting yield was significantly different from the biomass yield of the control culture (15.3 g_d.w._∙L^−1^). The lowest biomass concentration was noted at a selenium concentration of 30 mg Se^4+^∙L^−1^, and in the last day of *C. utilis* yeast culture, it amounted to 11.4 g_d.w._∙L^−1^ (Figure 1C).

There was no formation of red color in the cell biomass at 48-h culture. The red color indicates an occurrence of strong detoxification processes in the cell. Their mechanism in yeast cells in relation to selenite does not appear to be fully effective. On the one hand, it provides the reduction of selenium to elemental stage, and on the other hand, it generates oxidative stress with concurrent possibility of the depletion of the glutathione (GSH) pool, which is involved in detoxification processes [8]. The first stage in the change is a result of chemical reaction with the thiol groups of GSH. The resulting selenodiglutathione (GS-Se-SG)—characterized by high bioavailability—is subjected to the two-step enzymatic reduction first to glutathionylselenol (GS-SEH), and then to H_2_Se, which passively penetrating through vacuole membrane is returned to the cytoplasm, constituting the threat to the entire cell [8,16,17]. This may in turn cause a decrease in the biomass concentration compared to biomass obtained on the control medium.

Yang et al. [18] studied the effect of selenium on biomass yield of *C. utilis* SZU 07-01 yeasts. They performed a submersed culture in a biofermenter. They enriched the yeast cells in the synthetic medium supplemented with 15 mg Se^4^∙L^−1^ in two variants: (I) by the addition of selenium at the beginning of the culture, or (II) by the addition of selenium after 15 h during the logarithmic growth phase. According to their results, the biomass yield of the control culture was 13.2 g_d.w._∙L^−1^, whereas the biomass yield for variant I decreased by about 33% (8.9 g_d.w._∙L^−1^). In variant II, the addition of selenium at the 15-h culture did not adversely affect the growth of yeasts (13.1 g_d.w._∙L^−1^). Thus, it can be concluded that the toxicity of selenium for the yeast cells is highly dependent on the growth phase, and the cells in the adaptation phase are more sensitive to this microelement.

The presence of selenium in the experimental medium in addition to the prolonged time of culture (up to second day) affected the autolysis processes in yeast cells, which in turn resulted in the reduction in the yield of biomass. This could be due to the high chemical similarity of selenium and sulfur. Selenium undergoes a similar or identical metabolic pathway to sulfur in yeasts. It is a component of many proteins, which leads to the conformational changes in their functional activity [19]. These proteins may be subjected to irreversible changes in carbonylation processes, and reversible changes in the processes of post-translational modification (ubiquitination, phosphorylation, and sumoylation). In the presence of oxygen, selenite may favor the formation of reactive oxygen species (ROS), which consequently affects the DNA and possibly other cellular macromolecules [20].

### 2.2. Selenium Accumulation by Yeasts in Bioreactor Culture

The dynamics of selenium accumulation by yeasts is affected by the concentration and form of this element [21]. Having in mind the commonly low selenium content in food, one of the most economical sources of organic forms of selenium are yeast cells [22].

During a 24-h experimental culture in media supplemented with selenium at concentrations of 20 and 30 mg∙L^−1^, the content of this element permanently associated with the cell biomass of *C. utilis* yeast amounted to 427.5 and 442 μg∙g_d.w._^−1^. On the next day (after 48 h), the concentration of selenium was higher (1802 and 1841 μg∙g_d.w._^−1^). The observed course of selenium bioaccumulation in the cells showed that after the second day of culture, 61% to 90% of the initial content of selenium in experimental medium was bound with *C. utilis* yeasts.

Selenium may be subjected to biotransformation in the yeast cell structures (vacuole) to less toxic forms [23]. Yeasts also develop defense mechanisms involving the processes of the removal of H_2_Se formed in cellular structures. The resulting H_2_Se is also a major intermediate metabolite involved in the pathway of the synthesis of all forms of selenium produced in microbial cells [16]. Thus, it cannot be excluded that with an extending time of culture and an increase in selenium concentration in the culture medium, yeasts have developed mechanisms of tolerance that enable them to survive in unfavorable conditions.

Similar results were obtained by Kieliszek et al. [17]. They observed—in a laboratory scale (flat-bottom flasks) involving *C. utilis* ATCC9950 yeast cultures—that an increasing concentration of selenium in the experimental media caused an increase in the content of selenium in cell biomass. An efficient accumulation of selenium in yeast biomass was observed based on spectrophotometric method used for selenium determination with Variamine Blue [24]. *C. utilis* ATCC 9950 yeasts cultured in the presence of 20 mg Se^4+^ mg∙L^−1^ in the medium bound 500 µg Se^4+^∙g_d.w._^−1^ during the 24-h incubation. After the second day of growth, selenium content in the biomass increased to the level of 1500 µg Se^4+^∙g_d.w._^−1^. The highest content of selenium was noted in yeast biomass obtained from the medium supplemented with 60 mg Se^4+^∙L^−1^ for 72 h. However, the occurrence of red color of yeast cell biomass indicated an accumulation of elemental selenium in cell structures.

In this study, we observed that an increasing selenium concentration to the experimental medium caused an increase in selenium content in the yeast cell biomass of *C. utilis* ATCC 9950. A concentration of 30 mg∙L^−1^ of selenium in the culture medium fulfilled the expectations with respect to selenium content (1841 μg∙g_d.w._^−1^) as well as biomass yield (11.4 g_d.w._∙L^−1^) obtained after 48-h culture, which was lower compared to the control culture (14.9 g_d.w._∙L^−1^) (Figure 1C).

El-Nasser Khattab et al. [25] presented contradictory results from their study. They observed that *C. utilis* NRRL Y-184 yeast after third day of culture in the medium supplemented with 5 mg/L selenium(IV) oxide bound only 47.6% of selenium. A slightly lower level of selenium bioaccumulation (20.5%) in yeast cells was obtained in the medium containing 10 mg∙L^−1^. In the case of *S. cerevisiae* NRRL Y-139 yeast culture, the level of selenium binding from the culture medium (5 mg∙L^−1^) was 55%. An increase in the content of selenium(IV) oxide in experimental medium up to 10 mg∙L^−1^ caused a decrease in selenium bioaccumulation (24.5%) by the examined yeast strain.

Binding of selenium ions from the culture medium increases biosynthesis and secretion of chelating agents and ionophores by yeast cells, which leads to the breakdown of fatty acids and an increased permeability of cell membranes. Moreover, selenium accumulation in yeast cell biomass can affect the non-specific incorporation of selenium amino acids (e.g., SeMet) to the proteins [8,26].

Selenium binding by the yeast cells is reduced at high concentrations of sulfur and heavy metals in the culture medium [27]. This causes the accumulation of biologically inert elementary selenium [16], which does not have nutritive value. Therefore, the threshold of selenium toxicity in the culture medium depends on the yeast species, and tolerance for selenium may increase with its increasing concentration in the culture medium [8].

### 2.3. Selenite Transformation to SeMet in Yeast Cells

Biosynthesis of selenium amino acids in yeasts is similar to their sulfur counterparts (methionine, cysteine). In this aspect, the formation of selenium amino acids starts with *MET17* encoded homocysteine synthetase, connecting the previously formed H_2_Se with *O*-acethylhomoserine [26]. SeMet present in the largest quantities in the yeast cell is formed directly by homoselenocystein methylation. The reaction is performed by cobalamin-dependent homocysteine methyltransferase. After attaching to the tRNA^Met^, SeMet is used in translation [8].

**Figure 2:**
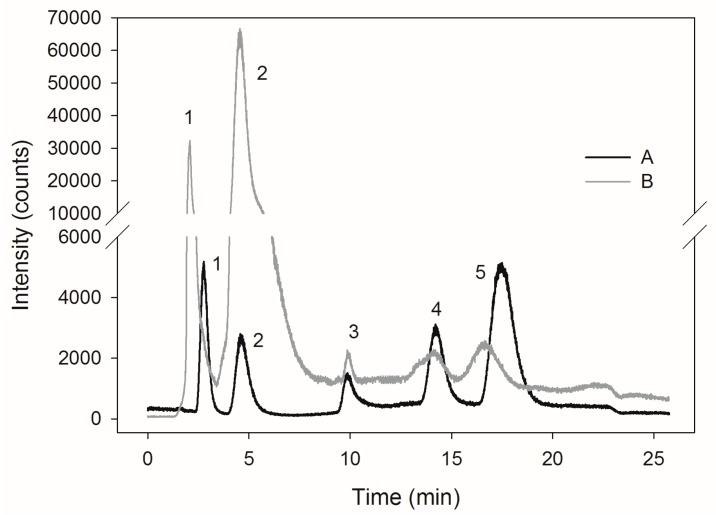
Chromatograms of a mixture of selenium compounds standards (A), reference material SELM-1 (B). The retention times were as follows: (1) seleno-methyl-selenocysteine, SeMetSeCys = 2.8 min, (2) selenomethionine, SeMet = 4.6 min, (3) γ-glutamyl-seleno-methyl-selenocysteine, γ-glutamyl–SeMetSeCys = 9.9 min, (4) selenite, Se(IV) = 14, (5) selenate, Se(VI) = 17.4 min.

Determination of SeMet content was verified using a commercial reference material SELM-1 (Figure 2A,B), in which the content of SeMet specified by the manufacturer was 3448 μg∙g_d.w._^−1^. In this study, we found 3021.94 μg∙g_d.w._^−1^. Analysis of a SELM-1 using this method resulted in 87.6% recovery of the certified value. McSheehy et al. [28] obtained a high content of SeMet (3256.9 μg∙g_d.w._^−1^) in selenium-enriched commercial yeasts using a mass spectrometer with ionization in inductively coupled plasma (ICP-MS). The total content of selenium in the preparation amounted to 2064.6 μg∙g_d.w._^−1^. The results were in good agreement with the certified values. Similar results concerning SeMet content (3461 μg∙g_d.w._^−1^) in SELM-1 preparation were obtained by Ward et al. [29]. The presence of a high concentration of SeMet in yeast cells indicates the presence of selenium in the organic form.

**Figure 3:**
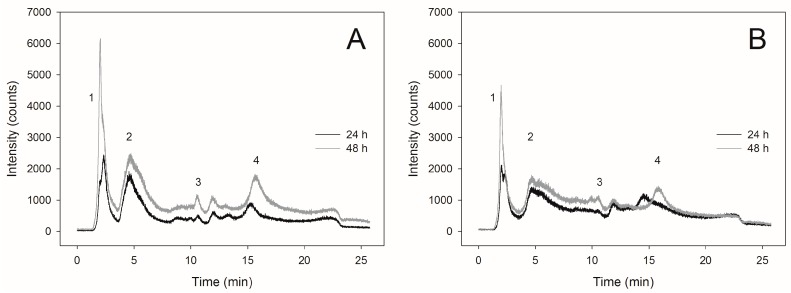
Chromatograms of selenium compounds separation obtained from *Candida utilis* ATCC 9950 yeasts biomass during 24- and 48-h culture on experimental media enriched with selenium at a concentration of (**A**) 20 mg∙L^−1^ and (**B**) 30 mg∙L^−1^. The retention times were as follows: (1) seleno-methyl-selenocysteine, SeMetSeCys = 2.8 min, (2) selenomethionine, SeMet = 4.6 min, (3) γ-glutamyl-seleno-methyl-selenocysteine, γ-glutamyl–SeMetSeCys = 9.9 min, (4) selenite, Se(IV) = 14, (5) selenite, Se(VI) = 17.4 min.

The highest content of SeMet in the cells of *C. utilis* ATCC 9950 yeasts (238.8 μg∙g_d.w._^−1^) was found after 48-h culture in the biomass obtained from the medium enriched with selenium at a concentration of 20 mg∙L^−1^ (Table 1). In the case of yeast obtained from the medium supplemented with 30 mg∙L^−1^, SeMet content in the biomass after 24-h culture was 42.8 μg∙gd_.w._^−1^. Extending the culture time to the second day, a slight increase in the content of SeMet (61.3 μg∙g_d.w._^−1^) was observed compared to the yeasts obtained after 24 h of culture. Therefore, the slight increase in SeMet content present in the reduced form was associated with the formation of its oxidized form. The presented chromatographic profiles (Figure 3A,B) demonstrate the occurrence of additional signals proving the formation of other selenium compounds.

Such low concentration of SeMet in *C. utilis* ATCC 9950 yeast could have been the result of the presence of high level of oxidized SeMet and other selenium compounds, inter alia, Se-methylselenocysteine. The presence of various selenium compounds in yeast cell biomass indicates the participation of that element in a variety of biochemical pathways that are associated with the protection of yeast cells against an excessive concentration of selenium in the culture medium. Depending on the yeast species, the level of SeMet in cell biomass may deviate from accepted standards, where 90% of the total selenium content in the yeast cells is present in the form of SeMet [1]. The rate of SeMet conversion to its oxidized form is quite difficult to predict, which in turn may lead to erroneous quantification results. The process of SeMet oxidation to oxidized SeMet (SeMetO) during sample preparation is one of the most important problems related to the precise determination of this compound’s levels. The degree of oxidation depends on the method of extraction and the temperature of sample storage [30]. Therefore, continuous development of new analytical techniques and improvement of the existing ones will enable the introduction of innovative methods allowing the identification of the forms in which selenium is subjected to biotransformation in yeast cells.

Using hydrophilic interaction liquid chromatography, Arnaudguilhem et al. [31] investigated the speciation of selenium in Sel-Plex (Alltech, Lexington, KY, USA) preparation, and noted the presence of 64 compounds containing selenium in their composition. Among these proteins, more than 50 were found to be selenium proteins. The highest number of selenium amino acids were incorporated into the heat shock proteins (HSPs) (SSA1 and SSA2 belonging to the family HSP70) and glyceraldehyde 3-phosphate dehydrogenase [12]. Using a semi-preparative liquid chromatography, McSheehy et al. [28] demonstrated the presence of about 30 selenium compounds isolated from the aqueous extracts of yeasts enriched with that element.

Ponce de León et al. [32] demonstrated contradictory results by examining the effect of enrichment of *S. cerevisiae* yeasts with selenium at different stages of the cell cycle. According to their results, the SeMet content in cell biomass did not increase, even when the total content of selenium stably associated with the yeast biomass was increased with an increasing concentration of selenium in the experimental medium. Analysis of the speciation of yeast biomass enriched with selenium (1825 μg∙g_d.w._^−1^) demonstrated the formation of a higher amount of l-SeMet (16 μg∙g_d.w._^−1^) in the exponential phase of cells growth, compared to the stationary phase (7 μg∙g_d.w._^−1^) with a total selenium content of 2114 μg∙g_d.w._^−1^.

A commercially available preparation SelenoExcell2 (Cypress Systems, Madera, CA, USA), which consists of selenium yeast, contains about 1250 μg∙g_d.w._^−1^ (±5%) selenium. This is a biological product, and the reproducibility of the results of SeMet determination may be varied [32,33]. This can be explained by the fact that the microorganisms—including different yeast species characterized by a different metabolism—may be capable of producing other organic compounds of selenium; for example, Se-methylselenocysteine.

From a nutritional point of view, SeMet is metabolized in the same way as its oxidized form. Thus, considering the oxidized form of that amino acid in the quantitative measurement of the total content of SeMet is fully justified [33].

## 3. Materials and Methods

### 3.1. Biological Material

In this study, we used *C. utilis* ATCC 9950 feed yeasts obtained from the collection of pure cultures of the Department of Biotechnology and Food Microbiology, University of Life Sciences in Warsaw.

### 3.2. Microbiological Media

Liquid YPD medium enriched with Na_2_SeO_3_ was used as an experimental medium for yeast-submerged cultures. The pH of the medium was adjusted to 5.0. The media and aqueous Na_2_SeO_3_ were sterilized at 121 °C for 20 min. Then, working solution of Na_2_SeO_3_ salt was added to the sterile YPD media in such volumes that the final selenium content in the experimental media was 20 and 30 mg∙L^−1^, respectively.

### 3.3. Yeasts Culture Using Bioreactor

Bioreactor cultures were performed in a 5-L BioFlo 3000 Bioreactor (New Brunswick Scientific, Edison, NJ, USA) with a working volume of 4 L. Inoculation of the experimental media in the bioreactor involved 40 mL of yeast cell suspension (7.0 × 10^8^ colony forming units (cfu∙mL^−1^) grown in inoculation culture (24 h). The media were oxygenated using compressed air (air flow at a rate of 0.7 vvm) until the relative solubility of oxygen in the medium (pO_2_) was 100%. The cultures were grown for 48 h at a temperature of 28 °C with a turbine stirrer speed of 400 rpm.

### 3.4. Determination of Cell Biomass Yield

Post-culture yield of yeast cell biomass was determined after centrifugation in a previously weighed thimble (3000× *g*, 10 min, 4 °C). Centrifuged cell biomass was dried at a temperature of 80 °C (SML 32/250 Zalmed, Warsaw, Poland) for 24 h to a constant weight. Biomass yield was expressed in grams of yeast dry weight (g_d.w._) per liter of culture medium (g_d.w._∙L^−1^ of medium).

### 3.5. Determination of Selenium Content in Yeast Cell Biomass

Culture medium for *C. utilis* yeast was centrifuged (3000× *g*, 10 min, 4 °C) and the supernatant was discarded. The resulting biomass was washed twice with deionized water, centrifuged, and then dried at 80 °C for 24 h until a constant weight. The samples (0.1 g_d.w._) of dried cell biomass were mineralized in 5 mL of 65% nitric acid in a microwave mineralizer (Multiwave Anton Paar, Graz, Austria) using a Teflon bomb (Anton Paar). Determination of the total selenium content (as isotope Se^82^) was performed using a quadrupole mass spectrometer with ICP-MS (Elan 6100 DRC, Perkin Elmer Sciex, Waltham, MA, USA).

ICP-MS allows the determination of total concentration of the selected element—that is, the total concentration of all forms of the element—which occurred in the examined solution [34,35]. The selenium content was verified on the reference material selenium enriched Yeast, certified reference material SELM-1 (National Research Council of Canada, NRC) with certified content of selenium: 2059 ± 64 μg∙g_d.w._^−1^ (National Research Council Canada, Institute for National Measurement Standards).

### 3.6. Determination of SeMet Content

SeMet content was determined in *C. utilis* yeast biomass after 24-h and 48-h culture. The culture medium was centrifuged for 10 min at 3000× *g* (Centrifuge Zalmed MPW-365, Warsaw, Poland), and then the supernatant was discarded. The resulting biomass was washed twice with deionized water and centrifuged again, and then dried at 80 °C (Centrifuge Zalmed SML 32/250, Warsaw, Poland) for 24 h to constant weight. Table 2 shows the system parameters used in the analysis.

The resulting dry yeast cell biomass (0.1 g_d.w._) was subjected to an extraction process involving enzymes protease (20 mg) and lipase (20 mg) in 5 mL of Tris-HCl 30 mmol∙mL^−1^ (pH 7.5) with an addition of 100 mL β-mercaptoethanol. The extraction was carried out in a thermostat stirrer for 16 h at a temperature of 37 °C, then the samples were centrifuged (13,000 × *g*, 10 min, 4 °C, Centrifuge MPW-365, Warsaw, Poland), and the resulting extract was filtered through a filter of pores diameter of 0.45 µm (Milex, France).

The selenium speciation in the extracts obtained was examined using high performance liquid chromatography (HPLC) ICP-MS technique. HPLC 1200 system (Agilent Technologies, Santa Clara, CA, USA) was combined with a quadrupole ICP-MS, model 6100 Elan DRC (Perkin Elmer Sciex). Separation of compounds using HPLC was performed using anion exchange column Hamilton PRP-X100 of the following dimensions: 250 mm × 4.1 mm × 10 μm (Hamilton, Reno, NV, USA). Ammonium acetate, 5 mmol∙mL^−1^ and 150 mmol∙mL^−1^, pH 4.7, was used as an eluent [34,35]. Standard solutions were prepared to obtain a concentration of 500 μg∙L^−1^ of selenium. Following were the retention times: SeMetSeCys = 2.8 min, SeMet = 4.6 min, γ-glutamyl–SeMetSeCys = 9.9 min, Se(IV) = 14, Se(VI) = 17.4 min (Figure 2A).

### 3.7. Statistical Analysis

The results were statistically analyzed using Statgraphics Plus 5.1 software (Statpoint Technologies, Warrenton, VA, USA). The significance of differences between mean values in a particular group was verified by Tukey test for the significance level of α = 0.05. Normality of the distribution of the analyzed parameters was evaluated using the Shapiro–Wilk test.

## 4. Conclusions

This study provides new information on the content of selenium during the growth of *C. utilis* ATCC 9950 yeast during the culture in a bioreactor. The process of yeast biomass enrichment with selenium is a natural method, which involves the biological potential of the cells to bioaccumulate selenium. For selenium yeasts production, the most effective is the short time of yeast culture up to 48 h, and selenium concentration at a level of 20–30 mg∙L^−1^. After 48 h of culture, a significant increase in selenium content in the yeast biomass was obtained (1841 μg∙g_d.w._^−1^). The highest content of SeMet (238.8 μg∙g_d.w._^−1^) was obtained from yeast biomass obtained after 48-h culture supplemented with 20 mg Se^4+^∙L^−1^. Due to an increased bioavailability of selenium present in the organic forms, it is necessary both as a biomedicine as well as a feed additive. These results show that yeasts have the ability to convert selenium present in the form of Na_2_SeO_3_ salt to its organic forms. A high degree of selenium binding with the yeast cells, as well as its intracellular biological transformations to organic forms, justify the possibility of using yeast cell biomass to produce supplements rich in selenium. This will compensate selenium deficiency in the diet of humans and animals.

## Figures and Tables

**Table 1:** The content of selenomethionine in *Candida utilis* ATCC 9950 yeast cell biomass.

Selenium Concentration in the Medium	Culvitation Time (h)	
	24 h	48 h
20 mg∙L^−1^	109.9 ± 5.62 *^,c^	238.8 ± 9.52 ^d^
30 mg∙L^−1^	42.8 ± 4.43 ^a^	61.3 ± 5.07 ^b^

* Means with the same letter a, b, c, d are not significantly different (acc. Tukey’s HSD test).

**Table 2:** Chromatographic separation procedure ICP-MS.

HPLC ICP-MS	
Column	Hamilton PRP–X100, 10 µm × 250 mm × 4.1 mm
Column temperature	23 °C
Flow	1 mL·min^−1^
Mobile phase	phase A: 5 mmol/mL ammonium acetate, pH 4.7 phase B: 150 mmol/mL ammonium acetate, pH 4.7
Elution program	0–4 min: 100% phase A 4–7 min: 0–100% phase B 7–25 min: 100% phase B
Split	100 μL
Detector	ICP-MS
Determined isotope	Se 82
Time of analysis	25 min

Abbreviations: ICP-MS, mass spectrometer with ionization in inductively coupled plasma; HPLC, high performance liquid chromatography.
